# Mining Mobile Network Fraudsters with Augmented Graph Neural Networks

**DOI:** 10.3390/e25010150

**Published:** 2023-01-11

**Authors:** Xinxin Hu, Haotian Chen, Hongchang Chen, Xing Li, Junjie Zhang, Shuxin Liu

**Affiliations:** 1 National Digital Switching System Engineering and Technological Research Center, Zhengzhou 450002, China; 2 The Edward S. Rogers Sr. Department of Electrical & Computer Engineering, University of Toronto, Toronto, ON M5S 3G4, Canada

**Keywords:** mobile network fraud, graph neural network, reinforcement learning, AdaBoost, graph imbalance

## Abstract

With the rapid evolution of mobile communication networks, the number of subscribers and their communication practices is increasing dramatically worldwide. However, fraudsters are also sniffing out the benefits. Detecting fraudsters from the massive volume of call detail records (CDR) in mobile communication networks has become an important yet challenging topic. Fortunately, Graph neural network (GNN) brings new possibilities for telecom fraud detection. However, the presence of the graph imbalance and GNN oversmoothing problems makes fraudster detection unsatisfactory. To address these problems, we propose a new fraud detector. First, we transform the user features with the help of a multilayer perceptron. Then, a reinforcement learning-based neighbor sampling strategy is designed to balance the number of neighbors of different classes of users. Next, we perform user feature aggregation using GNN. Finally, we innovatively treat the above augmented GNN as weak classifier and integrate multiple weak classifiers using the AdaBoost algorithm. A balanced focal loss function is also used to monitor the model training error. Extensive experiments are conducted on two open real-world telecom fraud datasets, and the results show that the proposed method is significantly effective for the graph imbalance problem and the oversmoothing problem in telecom fraud detection.

## 1. Introduction

**Figure 1:**
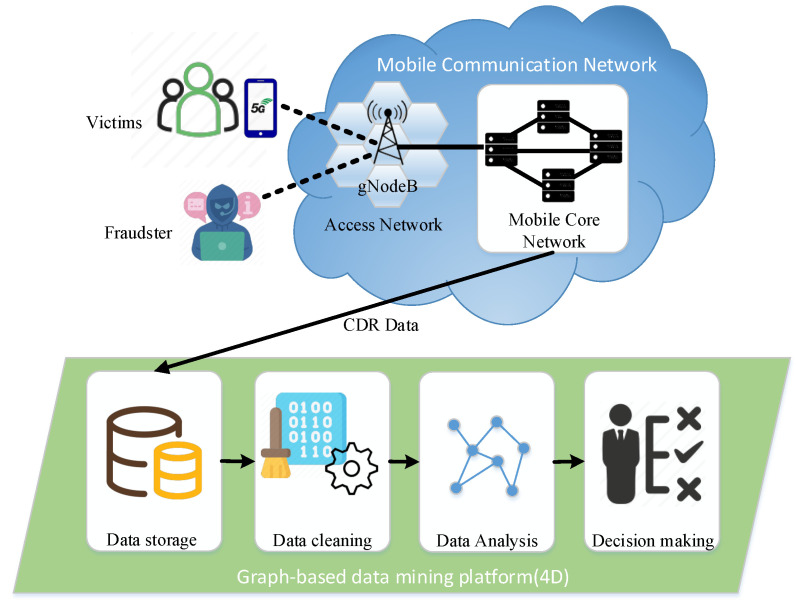
Illustration of fraud detection in mobile communication network.

In recent years, mobile network fraud, also known as telecom fraud, has become increasingly rampant around the world. In 2020, mainland China handled 230 million fraudulent calls and 1.3 billion fraudulent text messages. In the same year, one-third of the U.S. population suffered $19.7 billion in telecom fraud losses. Furthermore, over the past six years, U.S. telecom fraud has grown at an average annual rate of 30% [1]. In addition to affecting property safety, telecom fraud can also endanger victims’ lives (https://www.chinadaily.com.cn/china/2016-08/26/content_26600922.htm accessed on 29 November 2022). However, with the commercialization of 5G mobile networks, the number of fraudsters and victims is inevitably increasing even further. How to unearth telecom fraudsters has become an increasingly important research topic. In general, the process by which a fraudster commits a scam can be briefly described as follows. Fraudsters use especially designed scam scripts to lure victims into believing their false stories and transfer funds into designated accounts to obtain illicit income. While telecom fraud detection has attracted many efforts, the frequent changes in tactics, highly organized group collaboration, and actions of fraudsters deliberately disguised as ordinary users make it extremely difficult to detect fraudsters from large volumes of call detail records. Over the past few years, the advent of graph mining techniques has brought new possibilities for telecom fraud detection. This paper takes a 5G mobile communication network as an example to introduce how to use graph data mining technology to detect fraudulent users (see Figure 1). The subscribers (both fraudulent and benign subscribers) in the operator’s mobile network infrastructure generate a large number of CDRs every day. In addition, we use this data to create a graph of subscriber communication behavior, which can then be mined for fraudsters using graph machine learning techniques. We refer to the working steps of the graph-based data mining platform as 4D, namely Data storage, Data cleaning, Data analysis, and Decision making.

Previous works usually addressed telecom fraud detection for different behavioral characteristics or fraudster behavior patterns. For example, some studies regard the continuous behavior of users as behavior sequences, and use sequence learning methods to distinguish fraudsters from ordinary users [2]. There are also studies that regard fraud as the interaction between the fraudster and the victim, and some works try to solve the fraud detection of the network structure formed by the interaction [3,4]. In recent years, with the rise of graph machine learning technology, dealing with the fraud detection problem in a graph way has become one of the most mainstream methods [5,6,7,8].

However, there are two problems in current telecom fraud detection using graph machine learning techniques. One is that most of the work does not consider the problem of graph imbalance. The imbalance problem is common in anomaly detection research. However, its mirroring problem in graph data, the graph imbalance problem, is totally a novel one that is rarely noticed in graph machine learning related works [9], although the impact of graph imbalance on GNN model performance is significant. Specifically, fraudsters always represent a small fraction of the overall users, and the communication neighbors of fraudsters in a user communication network are always filled with a large number of benign users. This makes it extremely easy for the graph neural network model to discriminate fraudsters as benign users after message aggregation. In other words, the model is prone to bias against a large number of benign users. This problem is very detrimental to fraud detection, since detecting fraudsters is our real goal.

Another problem is the over-smoothing problem that is prevalent in GNN models. The over-smoothing effect is an important factor affecting the performance of GNNs [10]. Specifically, when the number of layers begins to increase, the performance of GNN begins to decline. This is because when the depth of the model is deepened, a small amount of useful information is easily overwhelmed by a large amount of noise, which eventually leads to the convergence of all node embeddings learned by the GNN model.

For the imbalance problem, we consider designing a reinforcement learning mechanism to reasonably sample the imbalanced graph, so that the balance of the graph can be optimized. Specifically, we first transform the node features based on the user communication behavior graph. Subsequently, a neighbor sampling mechanism based on reinforcement learning is designed, through which the optimal sampling probability can be learned to achieve similar neighbor sampling. In order to improve the performance of GNN and solve the problem of over-smoothing at the same time, we regard the GNN model as a weak classifier. We use the Adaboost algorithm to integrate multiple GNN models, and then learn to obtain the fused node embedding. Finally, the model training error is calculated using the class balanced focal loss function. Experiments on two real-world telecom fraud detection datasets show that the proposed method indeed has competitive performance.

The main contributions of this paper can be summarized as follows:

- We formulate the telecom fraud detection problem as node classification task and propose an augmented GNN framework to solve it.
- For the graph imbalance problem, we design a reinforcement learning-based graph sampling algorithm and train a semi-supervised model using class-balanced focal loss function.
- We treat GNNs as weak classifiers and integrate multiple GNNs to enhance their performance using the AdaBoost algorithm, while overcoming the oversmoothing problem of GNNs.
- Extensive experiments on two real-world telecom fraud detection datasets show that the method proposed in this paper is effective.

## 2. Related Work

### 2.1. Telecom Fraud Detection

Empirically, our work belongs to telecom fraud detection. To address the telecom fraud problem, early researchers adopted the rule-based approach [11,12,13]. They argue that different types of fraud have different fraud patterns. Therefore, domain experts can formulate rules for fraud detection.

This rule-based detection methods are effective, and highly interpretable. However, the development of detection rules is costly because it requires a lot of expert knowledge. In addition, fraudsters quickly change their tactics, which can easily invalidate established detection rules.

Considering the limitations of expert systems, follow-up works have begun to use classical machine learning methods to solve the problem of telecom fraud detection. These methods generally extract statistical features from raw CDR datas. These features are then fed into machine learning systems such as SVMs [14], decision trees and random forests [11,15], and Bayesian networks [16]. These methods can be divided into fully supervised, semi-supervised, and unsupervised according to whether labels are used or not. Among the fully supervised methods, there are decision trees, SVM, hidden Markovs, and neural networks. Unsupervised methods include density-based, distance-based, and cluster-based approaches. In recent years, attention mechanisms [5] and generative adversarial networks [17] have also been used in the field of telecom fraud detection.

These methods have greatly improved the detection effect, and can even detect unknown anomaly types. However, these methods ignore the natural connection relationship between users in the mobile network, such as user calls, text messages, and even contacts based on OTP applications. This kind of interaction information between users is very beneficial to the determination of fraudulent users.

### 2.2. Learning on Graph

Methodologically, our work belongs to graph-based machine learning. Early graph-based methods mainly include network representation learning methods based on graph topology, such as DeepWalk [18], Node2Vector [19], Line [20], and SDNE [21]. These methods are able to obtain a good low-dimensional embedding representation of nodes by the structure around the nodes and their positions in the graph. However, they only use the structural information of the graph and cannot effectively fuse the attribute information of the nodes.

Recently, important progress has been made in graph-based machine learning methods. For example, Kipf et al. proposed GCN [22], Velickovic et al. proposed GAT [23], and Hamilton et al. proposed GraphSAGE [24]. These methods adapt the well-performing deep neural network algorithms in computer vision to graph data, opening the door to graph deep learning. The models for fraud detection using graph neural networks can be roughly divided into three categories. The first category is GCN-based methods, such as GAS [25], Fdgars [26], GEM [27], iGAD [28], MDGCN [29], and AMNet [30], which personalize the GCN model for anomaly detection problems in different domains and achieve good results. The second category is GAT-based methods, such as SemiGNN [31], Player2Vec [32], Geniepath [33], and HACUD [34], which design practical semi-supervised anomaly detection methods with the help of attention mechanism. The third category is the methods based on GraphSAGE, such as GraphConsis [35], CARE-GNN [36], and PC-GNN [37], which implement inductive GNN anomaly detectors by adapting the GraphSAGE model. Most recently, unsupervised methods, such as graph contrastive learning, have also become increasingly popular for graph anomaly detection tasks [38,39,40,41,42,43,44]. These works use graph neural networks to efficiently solve financial fraud detection [25,26,27,31,33,34], and social networks fraud detection [32,35,36,37].

However, the above works do not touch the challenging area of telecom fraud, let alone the graph imbalance problem. In addition, due to the over-smoothing problem in graph neural networks, it is difficult to increase the number of layers effectively, making it hard to learn effective deep features. In this paper, we combine GNN and reinforcement learning for neighbor sampling in an attempt to solve the graph imbalance problem. Together with Adaboost, we treat GNNs as base classifiers and perform ensemble learning to overcome the over-smoothing problem.

## 3. Problem Definition

In this section, we introduce the definition of Graph imbalance problem, cost-sensitive learning, and GNN-based telecom fraud detection.

**Definition** **1.** ***Graph imbalance problem.** Given the labels Y of a set of nodes in a graph, there are m classes in total, namely $C=\{C_{1},\ldots ,C_{m}\}$. $∥C_{i}∥$ is the size of the i-th class.*

(1) $$IR=\frac{min_{i}(∥C_{i}∥)}{max_{i}(∥C_{i}∥)}$$

We use Equation (1) to measure the class imbalance ratio. Therefore, IR lies in the range [0,1]. If IR<1, then *C* is unbalanced. If IR=1, then *C* is balanced.

**Definition** **2.** ***Graph-based telecom fraud detection.** Taking subscribers in the mobile network as nodes, subscribers behavior as node features, and communication behavior between subscribers as edges to establish a graph, and then we can use GNN to detect fraudulent subscribers. Specifically, a user behavior graph G=(V,X,A,E,Y) in a mobile network is defined, where $V=\{v_{1},v_{2},v_{3},\ldots ,v_{N}\}$ is a set of nodes representing users in the mobile network. E represents the set of edges, that is, $E=\{e_{1},e_{2},e_{3},\ldots ,e_{M}\}$. $e_{j}=\left(v_{s_{j}},v_{r_{j}}\right)\in E$ is an edge between node $v_{s_{j}}$ and $v_{r_{j}}$, where $v_{s_{j}},v_{r_{j}}\in V$. $X=\{x_{1},x_{2},\ldots ,x_{N}\}$ is the set of original behavioral features of subscribers, where $x_{i}\in R^{d}$ is the behavioral feature vector of user $v_{i}$. Stacking these vectors into a matrix constitutes the feature matrix $X\in R^{N\times d}$ of the graph G. $A\in R^{N\times N}$ represents the adjacency matrix of graph G and $a_{i,j}=1$ means that there is an edge between node i and node j, if not, $a_{i,j}=0$. $Y=\{y_{1},y_{2},\ldots ,y_{N}\}$ is the set of labels corresponding to all nodes in the set V. For a given subscriber behavior graph, graph neural network can be used for node embedding representation learning. The inter-layer transfer formula in the GNN-based mobile fraud detection model can be formally described as:*

(2) $$h_{v}^{\left(l\right)}=\sigma \left(h_{v}^{\left(l-1\right)}⊕\mathrm{Agg}\left(\left\{h_{v^{\prime }}^{\left(l-1\right)}:\left(v,v^{\prime }\right)\in E\right\}\right)\right.$$

*where $h_{v}^{\left(l\right)}$ represents the embedding of node v at layer l, $h_{v}^{\left(0\right)}=x_{v}$, and $v^{\prime }$ is the neighbor of node v. Agg() is the message aggregation function, such as mean aggregation, pooling aggregation, attention aggregation, etc. *⊕* represents feature concatenation or summation operation.*

## 4. Proposed Method

**Figure 2:**
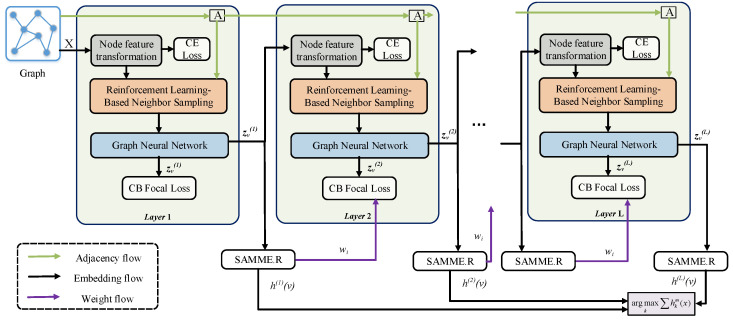
The pipeline of our proposed method.

The pipeline of our proposed method is shown in Figure 2. First, we input the original features of nodes into the fully connected layer for transformation. Then we feed the transformed graph and node embeddings into a well-designed reinforcement learning-based neighbor sampler to sample node neighbors based on node similarity. Subsequently, we use GNN to perform message aggregation on the filtered node neighbors and learn node embeddings. We regard the above process as a weak classifier, and use the ensemble learning method AdaBoost to integrate multiple weak classifiers to obtain the final embedding representation of each node. Finally, a balanced focal loss function is used to supervise the above training process.

### 4.1. Reinforcement Learning-Based Neighbor Sampler

To solve the graph imbalance problem, we consider using reinforcement learning techniques to sample the neighbors of each node, so that only some of the valid neighbors are retained. The whole process is divided into two steps: node feature transformation and neighbor sampling.

#### 4.1.1. Node Feature Transformation

In contrast to many unsupervised similarity metrics, practical problems (such as telecom fraud detection) usually require accurate data labels to assist in identifying fraudsters. For example, AGCN [45], DIAL-GNN [46], and NSN [47] adopt Gaussian kernel-normalized Mahalanobis distance, parameterized cosine similarity, and bilinear similarity-based inner product to measure similarity, respectively. However, these methods all have high time complexity. When dealing with graph representation learning problems in the real world, with a large number of nodes and edges, these methods consume a lot of time and computational resources.

Inspired by GraphMix [48] and CARE-GNN [36], we design a parametric node similarity metric that combines a fully connected network (FCN) and linear regularization. Specifically, we employ FCN as a node label predictor at each layer of the proposed model and use the Euclidean distance between the predictions of two nodes as a node similarity measure. For the embedding $h_{v}^{\left(l-1\right)}$ of the node *v* of the l−1th layer, the transformed embedding $h_{v}^{\left(l\right)}$ is expressed as follows:

(3) $$h_{v}^{\left(l\right)}=\sigma \left(Wh_{v}^{\left(l-1\right)}\right)$$

where σ is the activation function, which is implemented using ReLU. Furthermore, W is weight to be learned.

Then, when calculating the distance between an node *v* and its neighboring nodes $v^{\prime }$ at layer *l* of GNN, we take their embedding $h^{\left(l-1\right)}$ as input and use the Euclidean distance to calculate the following:

(4) $$D^{\left(l\right)}\left(v,v^{\prime }\right)={∥h_{v}^{\left(l\right)}-h_{v^{\prime }}^{\left(l\right)}∥}_{2}$$

Our goal is to make the high-dimensional feature distance of the transformed samples within the same class closer, and the different class farther away. To train the fully connected layers using the signals from the labels, we adopt the minimization of the cross-entropy loss function as the optimization objective:

(5) $$L_{simi}^{\left(l\right)}=-\sum_{v\in V}y_{v}\log\left(h_{v}^{\left(l\right)}\right)$$

#### 4.1.2. Neighbor Sampling

In the communication behavior graph, each node has a large number of neighboring nodes. However, these neighbors do not contribute equally to the determination of the central node class, and sometimes even interfere [36]. In telecom fraud detection, the impact of such a large number of invalid nodes on minority class samples is more obvious. This is because the neighbors of minority class nodes may contain a large number of majority class nodes, which directly affects the model’s discrimination of minority classes. This imbalance problem can be fatal to graph models. Because these models always tend to discriminate the minority class as the majority class, so that the model loss will be small. Therefore, we need to design a sampling mechanism to ensure that this adverse effect can be balanced. However, how to determine the reasonable sampling mechanism is a challenging problem.

Our starting point is to design an adaptive sampling criterion to automatically sample the optimal number of similar neighbors. Therefore, based on the neighbor similarity learning in the previous step, we uses top-p sampling with an adaptive filtering threshold to filter out similar neighbors that meet the requirements. To determine the optimal threshold, we also design a Reinforcement Learning (RL) algorithm based on Bernoulli multi-armed bandit (BMAB) during GNN training. Using the reward penalty mechanism of reinforcement learning, a most appropriate sampling probability *p* is automatically learned from the data. Then the neighboring nodes are ranked based on their similarity to the central node. Based on the similarity ranking result, for each node *v*, we sample its p*Degree(v) neighbors.

According to the Euclidean distance calculated in Equation (4), we can calculate the similarity between adjacent nodes *v*, $v^{\prime }$ as:

(6) $$S^{\left(l\right)}\left(v,v^{\prime }\right)=1-D^{\left(l\right)}\left(v,v^{\prime }\right)$$

The BMAB model can be expressed as B(S,A,f,T), where *S* is the state space, *A* is the action space, *f* is the reward and penalty function, and *T* is the termination condition. Given an initial sampling probability $p^{\left(l\right)}$, the agent chooses an action based on the current state, and the reward depends on the difference in the average similarity between two consecutive epochs. Next, we present the details of each BMAB component:

**State** To measure the sampling effectiveness of the neighbor sampler during training, we define a node-averaged similarity to portray the working state of BMAB. Specifically, the average similarity of nodes in the training set in the *l*-th layer GNN of the *e*-th epoch is defined as:

(7) $$G{\left(S^{\left(l\right)}\right)}^{\left(e\right)}=\frac{\sum_{v\in V_{\mathrm{train}\;}}S^{\left(l\right)}{\left(v,v^{\prime }\right)}^{\left(e\right)}}{∥E^{\left(l)(e\right)}∥}$$

where $∥E^{\left(l)(e\right)}∥$ is the number of edges in the training set $V_{\mathrm{train}\;}$. The numerator in the above equation represents the cumulative sum of similarity between each node and its neighbors in the training set, and the denominator represents the number of edges in the training set. A larger average similarity is our desired training goal, because the more similar the sampled neighbors and the central node are, the more valid the sampling result is. Therefore, we define the positive or negative difference in the average similarity of the nodes in two adjacent epochs as the system state, i.e., the state set $S=\{s_{1},s_{2}\}$, where state $s_{1}$: $G{\left(S^{\left(l\right)}\right)}^{\left(e-1\right)}<G{\left(S^{\left(l\right)}\right)}^{\left(e\right)}$, indicating that the average node similarity under the current epoch is greater than that of the previous epoch, and $s_{2}$: $G{\left(S^{\left(l\right)}\right)}^{\left(e-1\right)}\geq G{\left(S^{\left(l\right)}\right)}^{\left(e\right)}$, indicating that the average node similarity under the current epoch is less than or equal to the previous epoch.

**Action** The goal of the designed reinforcement learning module is to learn the optimal filtering threshold *p*. During the running of the model, BMAB needs to adjust the size of *p* according to the real-time state *s*. Here we give an action space $A=a_{1},a_{2}$, where action $a_{1}$: add fixed length τ to sampling probability *p*, $a_{2}$: subtract fixed length τ to sampling probability *p*.

For any state s∈S, the mapping relationship (policy) π between states and actions is defined as follows:

(8) $$a=\pi \left(s\right)=\left\{\begin{array}{cc} p+\tau , & s_{2}\\ p-\tau , & s_{1} \end{array}\right.$$

where τ is the change step size of neighbor sampling probability *p*, set manually as a hype-parameter of the model.

**Reward** Our starting point is that a suitable sampling probability *p* should ensure that a suitable number of neighboring nodes similar to the central node are sampled, while discarding neighboring nodes with large differences. Therefore, we design a reward mechanism. If the Agent increases the average similarity of nodes in the training set under $p^{\left(l\right)}$, it will be given a positive reward, otherwise it will receive a negative reward, i.e., a penalty. The formal description is as follows:

(9) $$f{\left(p^{\left(l\right)},a^{\left(l\right)}\right)}^{\left(e\right)}=\left\{\begin{array}{c} +1,\;G{\left(S^{\left(l\right)}\right)}^{\left(e-1\right)}\leq G{\left(S^{\left(l\right)}\right)}^{\left(e\right)}\\ -1,\;G{\left(S^{\left(l\right)}\right)}^{\left(e-1\right)}>G{\left(S^{\left(l\right)}\right)}^{\left(e\right)} \end{array}\right.$$

The reward is positive when the average distance of the newly selected neighbors of epoch *e* is smaller than the previous epoch, and vice versa. Estimating the cumulative reward is not easy. Therefore, we use immediate rewards to greedily update actions without exploration.

**Termination** Appropriate termination condition is crucial for Bernoulli multi-armed bandit to obtain the optimal threshold *p* and save computing resources. If the RL converges in the last 15 epochs, it indicates that an optimal threshold $p^{\left(l\right)}$ has been found and the sampler needs to be terminated. Here we give the formal termination condition as the following inequality:

(10) $$∥\sum_{e-15}^{e}f{\left(p^{\left(l\right)},a^{\left(l\right)}\right)}^{\left(e\right)}∥\leq 2,\;\mathrm{where}\;e\geq 15$$

It means that the sum of the rewards of 15 consecutive epochs of the RL-based neighbor sampler is less than or equal to 2 during the training process. To ensure that the sampler is trained for at least 15 rounds, we specify that e≥15. If inequality (10) is satisfied, then the sampler is converged. At this point, we obtain the optimal neighbor sampling probability *p*.

### 4.2. GNN-Based Weak Classifier

After neighbor sampling, node neighbor information needs to be aggregated. Often, we need to carefully design the aggregator to match the actual needs of the problem, which is what many popular GNN models do today. They make improvements to message passing and aggregation mechanisms to improve GNN performance. However, this method has limited improvement in the performance of GNN. Our idea is to treat ordinary GNN (such as GCN, GAT) as a weak classifier, and then use the ensemble learning (see Section 4.3) to improve the overall performance of GNN. Without loss of generality, we use Message Passing Neural Network (MPNN) [49] here to describe various types of GNNs uniformly. The forward pass of MPNN has two phases, a message passing phase and a readout phase. The message passing phase runs for *t* time steps and depends on the message function $M_{t}$ and the node update function $U_{t}$. During the message passing phase, the hidden state $h_{v}^{t}$ of each node is adjusted according to the message $m_{v}^{t+1}$.

(11) $$m_{v}^{t+1}=\sum_{v^{\prime }\in N\left(v\right)}M_{t}\left(h_{v}^{t},h_{v^{\prime }}^{t},e_{vv^{\prime }}\right)$$

(12) $$h_{v}^{t+1}=U_{t}\left(h_{v}^{t},m_{v}^{t+1}\right)$$

(13) $${\hat{y}}_{v}=R\left(\left\{h_{v}^{T}|vs.\in G\right\}\right)$$

where ${\hat{y}}_{v}$ is the label prediction result of node *v*. The message function $M_{t}$, the vector update function $U_{t}$ and the readout function *R* are all learnable differentiable functions.

In the model implementation (Section 5), we use a simple and effective aggregator, the average aggregator in GraphSAGE, for message aggregation. This method significantly reduces the model complexity compared with attention and other methods. Described as follows:

(14) $$h_{v}^{\left(t\right)}=\sigma \left(W\cdot \mathrm{MEAN}\left(\left\{h_{v}^{\left(t-1\right)}\right\}\cup \left\{h_{v^{\prime }}^{\left(t-1\right)},\forall v^{\prime }\in N\left(v\right)\right\}\right)\right.$$

where σ is the activation function. W is the weight to be learned, and N(v) is the neighbor of node *v*. MEAN() represents the operation of averaging the node embeddings in the set.

### 4.3. Ensemble GNN with SAMME.R

The original GAT model, as most GNN models, suffers from the over-smoothing problem, which is manifested as a rapid drop in performance as the number of GAT layers increases. An intuitive explanation is that after the central node aggregates the information from the multi-hop neighbors, the information of the node will be passed to the surrounding nodes in a random walk manner. Assuming that the message passes through infinite layers, the random walk distribution will converge to a stable value, making the obtained information too global and completely independent of the information of the central node itself. However, in many practical problems, such as fraud detection, we need to aggregate deeper features to better discover fraudsters. The challenge is that, for a given fraud detection dataset, it is difficult to know exactly how deep the model needs to be to achieve the best results. As can be seen in Section 5, the optimal number of GNN layers is different even on the two telecom fraud detection datasets. To address this issue, we consider combining the node embeddings obtained by each GNN weak classifier in the previous section with the SAMME.R algorithm [50] for ensemble learning. Specifically, we assign different weights to each GNN weak classifier, and finally integrate the node embeddings learned by multiple GNNs to obtain the final embedding of the model.

Consider the *l*-th layer (*l*-th GNN) of the proposed method as the *l*-th base classifier. After the final embedding $z_{v}^{\left(l\right)}$ of node *v* is obtained in the *l*-th GNN, we normalize $z_{v}^{\left(l\right)}$ by the softmax function to obtain the probability that node *v* belongs to each class in the *l*-th weak classifier.Thus, the probability vector of node *v* being classified into *k* different categories can be expressed as:

(15) $$\begin{array}{cc} p^{\left(l\right)}\left(v\right) & =\left[p_{1}^{\left(l\right)}\left(v\right),\ldots p_{k}^{\left(l\right)}\left(v\right),\cdots p_{K}^{\left(l\right)}\left(v\right)\right]\\ \; & =\mathrm{Softmax}\left(z_{v}^{\left(l\right)}\right) \end{array}$$

Assuming that the first (l−1)th base classifier is $f^{\left(l-1\right)}\left(vs.\right)$, we want to add a new base classifier $h^{\left(l\right)}\left(v\right)$ to make the overall model perform better. According to the output of the *l*-th GNN, combined with the SAMME.R algorithm, the base classifier $h^{\left(l\right)}\left(x\right)$ at the *l*-th layer can be calculated as:

(16) $$h_{k}^{\left(l\right)}\left(v\right)=\left(K-1\right)\left(\log p_{k}^{\left(l\right)}\left(v\right)-\frac{1}{K}\sum_{k^{\prime }}\log p_{k^{\prime }}^{\left(l\right)}\left(v\right)\right),k=1,\ldots ,K$$

In order to make the misclassified nodes in $h^{\left(l\right)}\left(x\right)$ obtain a greater probability of being correctly classified in the (l+1)th base classifier, the weights of the nodes need to be updated:

(17) $$w_{v}^{\left(l+1\right)}=w_{v}^{\left(l\right)}\cdot \exp\left(-\frac{K-1}{K}y_{v}\log p^{\left(l\right)}\left(v\right)\right),vs.\in V$$

where $w_{v}^{\left(l+1\right)}$ represents the weight of node *v* in the (l+1)th base classifier, and p(v) is calculated by Equation (15). The base classifiers h(v) of each layer are combined to obtain the ensemble classification results as follows:

(18) $$C\left(v\right)=\arg\max_{k}\sum_{l=1}^{L}h_{k}^{\left(l\right)}\left(v\right)$$

### 4.4. Proposed Algorithm

Loss function is crucial to the imbalanced learning. However, the widely used cross-entropy loss function performs poorly on extreme imbalanced problems. For the graph imbalance problem, we borrow the class-balanced focal loss [51] in the field of computer vision to constrain the training process of the proposed model.

(19) $$L_{GNN}^{\left(l\right)}=-\frac{1-\beta }{1-\beta^{n_{y}}}\sum_{i=1}^{C}{\left(1-p_{i}^{t}\right)}^{\gamma }\log\left(p_{i}^{t}\right)$$

where the hyperparameter β∈[0,1) controls how fast effective number grows as *n* increases, $n\in Z_{>0}$ is the number of samples, γ≥0 is a tunable focusing parameter.

The complete train processing of the proposed model is shown in Algorithm 1.
**Algorithm** **1:** Augmented Graph Neural Networks.
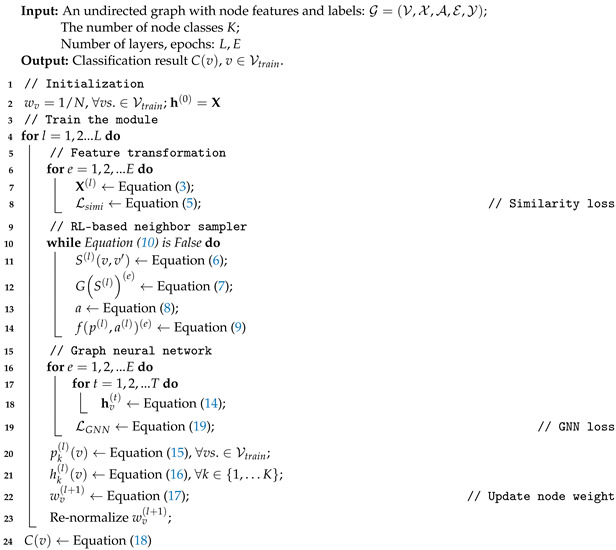


## 5. Experiments

This section will present the performance of the proposed method on two real-world telecom fraud detection datasets. It mainly includes the following aspects:

- Comparisons of the proposed method with state-of-the-art methods in graph-based anomaly detection
- The effectiveness of the proposed method on solving graph imbalance problem
- The effectiveness of the proposed method on overcoming the GNN over-smoothing problem

### 5.1. Experimental Setup

#### 5.1.1. Dataset

This paper uses two real-world imbalanced telecom fraud detection datasets, Sichuan and BUPT.

Sichuan: This dataset contains CDR data of 6106 users in 23 cities in Sichuan, a large Chinese province, with a time span from August 2019 to March 2020 [6]. The node features come from the feature extraction of user behavior records, and the edges are constructed by Hu et al. [6] based on the node feature similarity. All samples contain a total of two classes, namely fraudsters and the normal. In this dataset, the imbalance rate IR=1962/4144=0.4735.

BUPT: This dataset includes a week’s CDR data of users in a Chinese city [5]. The author extracts features from the original CDR data, and establishes connections based on user communication behaviors. The dataset contains three classes, in which the data imbalance rate IR=8074/99861=0.0809, the details are shown in Table 1.

#### 5.1.2. Baseline Method

To verify the effectiveness of the proposed method, we compare it with various GNN baselines in a semi-supervised learning setting. Specifically, we chose GCN [22], GAT [23], and GraphSAGE [24] as general GNN models. We choose FdGars [26], GraphConsis [35], GEM [27], SemiGNN [31], and BTG [6] as state-of-the-art GNN-based fraud detectors. Since our graph is a homogeneous graph, all the above methods are computed in a homogeneous graph manner.

- GCN: GNN that aggregates neighbor information by spectral graph convolution
- GAT: A GNN that uses an attention mechanism to aggregate neighbor node information
- GraphSAGE: An inductive GNN with a fixed number of sampled neighbors
- FdGars: A GCN-based Social Opinion Fraud Detection System
- GraphConsis: A Heterogeneous Graph Neural Network for Graph Inconsistency
- GEM: A Heterogeneous Graph Fraud Detection GNN Based on Attention Mechanism
- SemiGNN: A GNN for Hierarchical Attention Aggregation with Multi-view
- BTG: A GNN-based Telecom Fraud Detector for Sparse Graphs

#### 5.1.3. Evaluation Metrics and Experiment Settings

For the evaluation of imbalanced problems, the evaluation indicators are very critical. In order not to be biased, we use four widely used indicators to measure the performance of all comparison methods: Macro AUC, Macro recall, Accuracy, and Marco F1.

**AUC** is the area under the ROC curve, which is defined as:

(20) $$\mathrm{AUC}=\frac{\sum_{u\in U^{+}}rank_{u}-\frac{∥U^{+}∥\times \left(∥U^{+}∥+1\right)}{2}}{∥U^{+}∥\times ∥U^{-}∥}$$

where $U^{+}$ and $U^{-}$ indicate the minority and majority class set in the testing set, respectively. Furthermore, $rank_{u}$ indicates the rank of node *u* via the score of prediction.

**Recall** metric is very important for the imbalance problem, which can accurately measure the proportion of a few important categories that are detected. It is defined as follows:

(21) $$\mathrm{Recall}=\frac{\mathrm{TP}}{\mathrm{TP}+\mathrm{TN}}$$

where TP and TN represent the number of true positive and true negative samples in the confusion matrix, respectively. Macro recall is the arithmetic mean of multiple classes, which treats all classes equally, regardless of the importance of different classes.

**Accuracy** is the ratio of the number of correctly classified samples to the total number of samples, which is defined as:

(22) $$\mathrm{Accuracy}=\frac{\mathrm{TP}+\mathrm{TN}}{\mathrm{All}}$$

**F1** is another comprehensive indicator for evaluating imbalanced problems, which is defined as follows:

(23) $$F1=2\frac{\;\mathrm{precision}\;\times \;\mathrm{recall}\;}{\;\mathrm{precison}\;+\;\mathrm{recall}\;}$$

where macro-f1 is to calculate the arithmetic mean of F1 scores for each class.

In the above metrics, the higher the score means the better the performance of the model. In the experiments, we randomly select training samples and keep the ratio of positive and negative samples in the training set the same as the whole dataset. For our proposed method, we use Adam optimizer for parameter optimization in the experiments, and the specific configuration is as follows. For the both of the datasets, we set hid embedding size (64), learning rate (0.01), model layers (2), train size (0.2), test size (0.6), max epoch (30), and γ=2, α=1. We implement the proposed method by Pytorch; all models are running on python3.7.10, 1 GeForce RTX 3090 GPU, 64GB RAM, 16 cores Intel(R) Xeon(R) Gold 5218 CPU @2.30GHz Linux Server.

### 5.2. Overall Evaluation

During the experiment, we set the training size:validation size:test size as 0.2:0.2:0.6. We obtain the scores of the baseline methods and the proposed model on four evaluation metrics as shown in Table 2. It can be observed that our proposed method outperforms the comparative methods on all metrics on two real-world telecom fraud detection datasets. From the experimental results, we observe that general GNNs such as GCN, GAT, and GraphSAGE outperform GNN-based fraud detectors such as FdGars, GraphConsis, GEM, and SemiGNN on two telecom fraud datasets. The reasons for the above phenomenon may be threefold. First, the general GNN is universal. GCN, GAT, and GraphSAGE are early works in the field of GNN. Their design starting point is to perform deep learning of graphs for general graphs. The design idea is very simple, which directly leads to the excellent performance in graph deep learning. Second, GNNs designed for problems in different domains have large variability in performance on the same problem. Even though they are all fraud detectors, they focus on different domains such as social, finance, insurance, and gaming, and thus have different characteristics, which directly leads to detectors that excel in this domain to perform mediocre in other domains. FdGars, GraphConsis, GEM, and SemiGNN are, respectively, designed to solve problems in different fields such as social fraud and financial fraud. However, their performance in telecom fraud detection is not as good as general GNN and telecom fraud detector BTG. Third, the performance of fraud detectors designed for different types of graphs (such as homogeneous graphs and heterogeneous graphs) is also very different. FdGars, GraphConsis, GEM, and SemiGNN are fraud detectors designed for heterogeneous graphs, and the telecom fraud detection dataset used in this paper is a homogeneous graph, which may also lead to their performance degradation. This further illustrates that the fraud detection method on the graph has strong scenario dependence. In addition, it should be noted that the BTG detection framework for telecom fraud has better performance on the two telecom fraud detection data sets, but it does not take into account the graph imbalance problem and the natural over-smoothing problem of the GNN model. Its performance, although better than the generic GNN and GNN-based fraud detector, is still lower than the approach proposed in this paper.

### 5.3. Alleviation of Graph Imbalance Problem

**Figure 3:**
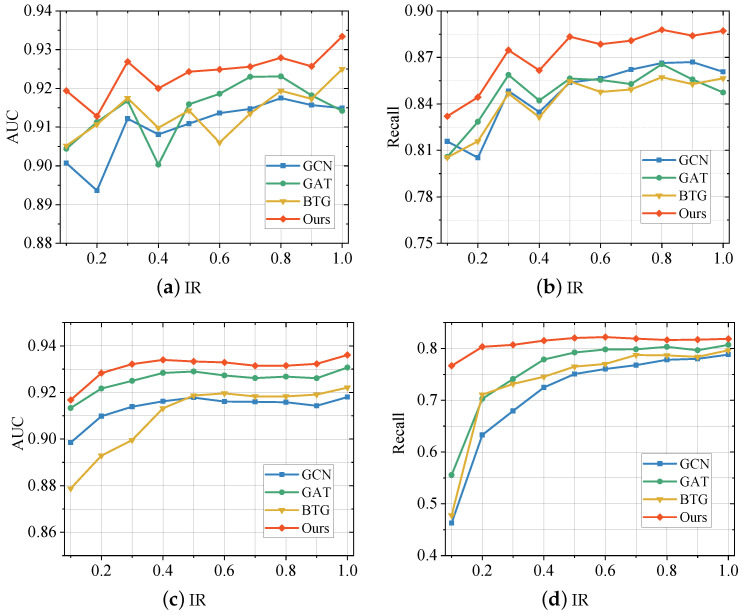
Performance Comparison between the proposed method and baselines with different imbalance rate (IR) on Sichuan Dataset (**a**,**b**) and BUPT Dataset (**c**,**d**).

To verify the effectiveness of the proposed model for the graph imbalance problem, we illustrate it by setting up a set of experiments. Specifically, we randomly sample samples from the two telecom fraud datasets used in this paper according to varying IRs (ranging from 0.1 to 1), thereby generating new datasets that conform to the IRs. Several of the best performing baseline methods (GCN, GAT, BTG) in Section 5.2 were then selected for comparison with the proposed method in this paper, and their scores on both AUC and recall are plotted in the curves shown in Figure 3.

From the figure, we can observe that the method proposed in this paper outperforms the baselines at various imbalance rates. When the IR decreases, the scores of all models start to decrease, but the method proposed in this paper decreases more slowly. In addition, the classic GNN models, GCN and GAT, also have good performance. However, when the IR decreases, the performance of these models still degrades faster than the method proposed in this paper. The same goes for the telecom fraud detector BTG. It is worth noting that BTG, as a detection model especially designed for telecom fraud, has better overall performance, but its performance on imbalanced datasets is still weaker than the method proposed in this paper. This corroborates the important role played by the particular design of the proposed method for the imbalance problem. In particular, the reinforcement learning-based neighbor sampling strategy balances the data distribution and the supervisory role of class-balanced focal loss in the model training process.

Furthermore, we observed that all methods differ in their sensitivity to data imbalance on the two data sets. Specifically, these GNN models are more sensitive to the imbalance of BUPT, i.e., the performance of the fraud detector drops significantly when the IR decreases. On the Sichuan dataset, the performance of various comparison methods decreases slowly as the IR decreases. One possible reason is that the average degree of nodes in the Sichuan dataset is much larger than that of BUPT, which alleviates the data imbalance. Nevertheless, the proposed method still outperforms other methods, which indicates that the imbalanced design in the model is effective.

### 5.4. Avoidance of Over-Smoothing Effects

**Figure 4:**
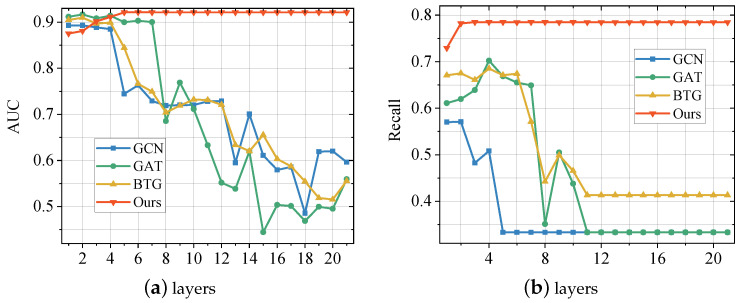
Performance Comparison between the proposed method and baselines with different GNN layers on BUPT Dataset. (**a**) illustrates the performance score of the models on metric AUC; (**b**) illustrates the performance score of the models on metric Recall.

To verify the effect of our proposed method on the GNN oversmoothing problem, we compare the performance of the proposed method with GCN, GAT, and BTG on AUC and recall. In order not to take up too much space, we conducted experiments only on the BUPT dataset. We change the number of neural network layers (1–21) and the rest of the hyperparameters are configured the same as in Section 5.1.3. The obtained experimental results are shown in Figure 4. From the result, we can observe that GCN, GAT, and BTG are obviously affected by the over-smoothing effect. As the number of network layers increases, the performance of the model on all two metrics, AUC and recall, degrades significantly. In contrast, the scores of the method proposed in this paper first rise slowly and then remain stable. This shows that our proposed method can effectively avoid the over-smoothing effect of GNN. The reason for this phenomenon is that we ensemble multiple GNN models using the SAMME.R algorithm, thus avoiding the effect of over-smoothing.

## 6. Conclusions

This paper investigates the GNN-based telecom fraud detection and two problems associated with it, namely, graph imbalance and over-smoothing. To solve them, we combine reinforcement learning and ensemble learning with GNN, and propose an augmented GNN model. First, we design a reinforcement learning mechanism for node neighbor sampling in the graph. Then, all the nodes perform representation learning on the sampled graph with GNN aggregator. To solve the over-smoothing problem, we consider GNN models as base classifiers and use AdaBoost to integrate multiple GNNs. Finally, the entire training process is supervised using a class balanced focal loss function. Extensive experiments on two real-world telecom fraud datasets demonstrate the effectiveness of the proposed method. Furthermore, it is found that the reinforcement learning-based neighbor sampler is beneficial to overcome the graph imbalance, and the Boosting architecture is helpful for the oversmoothing problem of GNN. The augmented GNN proposed in this paper can be applied not only to telecom fraud detection, but also to social network fraud detection, financial fraud detection, cyber security, and other fields where graph data imbalance problem exists.

## Figures and Tables

**Table 1:** Dataset and graph statistics.

Dataset	Nodes (Fraud Ratio)	Edges	Classes	Features	IR
**Sichuan**	6106 (32.1%)	838,528	Benign: 4144	55	0.4735
			Fraud: 1962		
**BUPT**	116,383 (7.3%)	350,751	Normal: 99,861	39	0.0809
			Fraudster: 8448		
			Courier: 8074		

**Table 2:** Performance comparison on two real-world telecom fraud datasets.

Dataset	Metric	GCN	GAT	Graph-Sage	FdGars	Graph-Consis	GEM	SemiGNN	BTG	Ours
**Sichuan**	**Macro AUC**	0.9163	0.9146	0.9159	0.7887	0.7615	0.8019	0.6958	0.9183	**0.9312**
	**Macro recall**	0.8547	0.8510	0.8564	0.7082	0.6865	0.7230	0.5820	0.8426	**0.8824**
	**accuracy**	0.8812	0.8797	0.8829	0.7325	0.7238	0.7950	0.6113	0.8881	**0.9155**
	**Macro F1**	0.8645	0.8519	0.8631	0.6499	0.6853	0.7401	0.5643	0.8629	**0.8933**
**BUPT**	**Macro AUC**	0.8932	0.8686	0.8928	0.6462	0.6294	0.6346	0.5732	0.8952	**0.9016**
	**Macro recall**	0.5706	0.5573	0.6715	0.4357	0.3319	0.3344	0.4007	0.6731	**0.7281**
	**accuracy**	0.9008	0.8875	0.9113	0.8651	0.8446	0.8583	0.8237	0.9076	**0.9250**
	**Macro F1**	0.6265	0.5835	0.6918	0.4027	0.3127	0.3100	0.3514	0.6692	**0.7395**

## Data Availability

The datasets that support this study are available from https://aistudio.baidu.com/aistudio/datasetdetail/40690 (accessed on 1 September 2022) and https://github.com/khznxn/TF-Dataset (accessed on 1 September 2022).
